# Retraction Note: Synergistic effects of combinatorial chitosan and polyphenol biomolecules on enhanced antibacterial activity of biofunctionalized silver nanoparticles

**DOI:** 10.1038/s41598-025-30800-0

**Published:** 2025-12-16

**Authors:** Niloufar Hajarian Rezazadeh, Foad Buazar, Soheila Matroodi

**Affiliations:** 1 Department of Marine Chemistry, Khorramshahr University of Marine Science and Technology, PO. Box 669, Khorramshahr, Iran; 2 Department of Marine Biology, Khorramshahr University of Marine Science and Technology, PO. Box 669, Khorramshahr, Iran

Retraction of: *Scientific Reports* 10.1038/s41598-020-76726-7, published online 12 November 2020

The Editors have retracted this Article.

After publication, concerns were raised about image duplication of AgNP tubes in Figure 1. In addition, concerns relating to the veracity and analytical interpretation of spectral data in Figures 2, 5 and 6 were also raised. The Authors did not respond to the Editors requests to provide the original images and raw data for verification. The Editors have therefore lost confidence in the veracity of the data presented.

Soheila Matroodi disagrees with this retraction. Niloufar Hajarian Rezazadeh and Foad Buazar did not respond to the correspondence from the Editors about this retraction.

